# Integrated Lung and Tracheal mRNA-Seq and miRNA-Seq Analysis of Dogs with an Avian-Like H5N1 Canine Influenza Virus Infection

**DOI:** 10.3389/fmicb.2018.00303

**Published:** 2018-03-05

**Authors:** Cheng Fu, Jie Luo, Shaotang Ye, Ziguo Yuan, Shoujun Li

**Affiliations:** ^1^College of Veterinary Medicine, South China Agricultural University, Guangzhou, China; ^2^Guangdong Provincial Key Laboratory of Prevention and Control for Severe Clinical Animal Diseases, Guangzhou, China; ^3^Guangdong Technological Engineering Research Center for Pet, Guangzhou, China

**Keywords:** canine influenza virus, H5N1, mRNA–miRNA integrate analysis, negative, KEGG

## Abstract

Avian-like H5N1 canine influenza virus (CIV) causes severe respiratory infections in dogs. However, the mechanism underlying H5N1 CIV infection in dogs is unknown. The present study aimed to identify differentially expressed miRNAs and mRNAs in the lungs and trachea in H5N1 CIV-infected dogs through a next-generation sequencing-based method. Eighteen 40-day-old beagles were inoculated intranasally with CIV, A/canine/01/Guangdong/2013 (H5N1) at a tissue culture infectious dose 50 (TCID_50_) of 10^6^, and lung and tracheal tissues were harvested at 3 and 7 d post-inoculation. The tissues were processed for miRNA and mRNA analysis. By means of miRNA-gene expression integrative negative analysis, we found miRNA–mRNA pairs. Lung and trachea tissues showed 138 and 135 negative miRNA–mRNA pairs, respectively. One hundred and twenty negative miRNA–mRNA pairs were found between the different tissues. In particular, pathways including the influenza A pathway, chemokine signaling pathways, and the PI3K-Akt signaling pathway were significantly enriched in all groups in responses to virus infection. Furthermore, dysregulation of miRNA and mRNA expression was observed in the respiratory tract of H5N1 CIV-infected dogs and notably, TLR4 (miR-146), NF-κB (miR-34c) and CCL5 (miR-335), CCL10 (miR-8908-5p), and GNGT2 (miR-122) were found to play important roles in regulating pathways that resist virus infection. To our knowledge, the present study is the first to analyze miRNA and mRNA expression in H5N1 CIV-infected dogs; furthermore, the present findings provide insights into the molecular mechanisms underlying influenza virus infection.

## Introduction

Although the natural host of influenza A virus is wild aquatic birds, the host barrier is not unbreakable, and the virus can be transmitted to other species, including dogs (Klenk, 2014). Canine influenza virus (CIV) had not been reported until 2004, and it was first discovered in racing Greyhounds in the United States. The first identified CIV was confirmed as subtype H3N8 by sequencing (Crawford et al., 2005). In addition, another subtype of CIV (H3N2) was isolated from sick farmed and pet dogs in South Korea (SK) in 2007 (Lee et al., 2009; Li et al., 2010). Moreover, multiple subtypes of influenza A viruses are reported to infect dogs, including the deadly H5N1 avian influenza virus (Songserm et al., 2006b), influenza A (H1N1) pdm09 virus (Damiani et al., 2012; Su et al., 2014a,b; Yin et al., 2014), H10N8 avian influenza virus (Su et al., 2014b), avian-like H9N2 influenza A virus (Songserm et al., 2006a; Sun et al., 2013), avian-like CIV (Su S. et al., 2013), and H3N2 influenza virus with the PA gene of H9N2 avian influenza virus (Lee et al., 2016). Studies have reported successful experimental infection of CIV in guinea pigs and mice (Tu et al., 2009; Castleman et al., 2010). These findings indicate that dogs may represent a new bridging species for avian and human influenza viruses for interspecies transmission.

In October 2004, highly pathogenic avian influenza (HPAI) H5N1 virus was reported for the first time in Thailand in a dog with severe lung congestion and edema and bloody nasal discharge (Songserm et al., 2006b). Furthermore, previous studies have reported that H5N1 influenza virus infections in dogs have resulted in anorexia, fever, conjunctivitis, labored breathing, and coughing (Songserm et al., 2006b; Maas et al., 2007; Chen et al., 2010; Ashour et al., 2012). Being considered “man’s best friend,” dogs drastically influence human lives. However, influenza viruses are susceptible to mutation; hence, it is important to understand the pathogenesis of H5N1 influenza virus infection among dogs.

MiRNAs are small non-coding RNAs. MiRNA binds on target mRNA to negatively regulate biological processes such as differentiation (Shivdasani, 2006), proliferation (Song et al., 2010), growth (Suh et al., 2015), metabolism (Gomez et al., 2014; Meydan et al., 2016), and apoptosis (Cheng, 2005). Increasing evidence has shown that deregulation of miRNA contributes to antiviral activity during an influenza virus infection. MiR-342-5p participates in macrophage interferon (IFN) antiviral responses against multiple viruses including influenza A (H1N1) (Ploegh et al., 2016). MiR-146a up-regulation significantly decreases H1N1 and H3N2 viral propagation (Terrier et al., 2013). MiR-144 was reported to post-transcriptionally decrease *TRAF6* levels to attenuate the antiviral response (Lopez et al., 2017). MiR-485 binds *PB1* transcripts in H5N1 to inhibit viral replication (Ingle et al., 2015). These findings suggest that certain miRNAs may play an important role in regulating influenza virus infections in dogs.

Strikingly, recent studies have assumed that miRNAs not only develop functions but also transmit from one species to another, thereby promoting crosstalk (Zhang et al., 2016) and interfering in signal transmission and communication (Hansen et al., 2010). Numerous studies have reported that viruses adapt their own miRNAs based on host miRNA (Pfeffer et al., 2004; Cai et al., 2005; Pfeffer et al., 2005) and establish an environment conducive to viral replication (Grey et al., 2007; Samols et al., 2007; Stern-Ginossar et al., 2007; Choy et al., 2008; Murphy et al., 2008; Nachmani et al., 2009). Hence, understanding the mechanism underlying viral miRNA-mediated adaptations can further our knowledge of the cross-species communication.

When infected with a virus, animals try to defend themselves with transcriptional reprograming of the affected cells. In this process, several genes are key elements and these genes are regulated by miRNAs (Peng et al., 2011; de Cubas et al., 2013). Recently, next-generation sequencing (NGS) technology has been used to obtain comprehensive sequencing data, which was used to detect and study the miRNA and protein expression levels in dogs with influenza virus infection (Zhao et al., 2014; Su et al., 2015). However, no detailed analysis of miRNA and the mRNA transcriptome is available. In this study, we used miRNA and mRNA profiles to perform a deep analysis of critical genes, miRNAs, and pathways related to virus infections.

## Materials and Methods

### Sample Collection and RNA Isolation

Canine influenza virus, A/canine/01/Guangdong/2013 (H5N1), was isolated in 2013 from a dog with severe respiratory symptoms. Eighteen 40-day-old beagles were assigned to experimental and control groups. Dogs were housed in the Laboratory Animal Center of South China Agricultural University with number SYXK (YUE) 2014-0136. All study protocols were approved by the ABSL-3 Committee of South China Agricultural University in this study. A hemagglutination inhibition (HI) assay revealed that these dogs were sero-negative for avian-origin CIV (H3N2 and H5N1) and for H1N1, H3N1, and influenza B viruses of seasonal influenza viruses. Nine beagles were randomly divided into each group, i.e., experimentally infected (I) and non-infected (NI). After beagles were anesthetized with tiletamine–zolazepam (Virbac, 10–15 mg/kg), they were inoculated intranasally with H5N1 CIV at a tissue culture infectious dose 50 (TCID_50_) 10^6^ and the control group similarly received 1.0 ml of phosphate-buffered saline. Nasal samples were collected from all beagles before infection and continuously for 14 d after infection. At 3 and 7 d post-infection (dpi), three beagles from each group were euthanized through a pentobarbital overdose. Lesions in the lungs and trachea were collected and frozen in liquid nitrogen. One section was immediately used for RNA isolation, and the others were stored at -80°C for further use. An RNA library was generated for each group from the total RNAs collected from three dogs. Total RNAs were isolated using TRIZOL (Takara, Otsu, Japan) in accordance with the manufacturer’s protocol. The concentration and purity of RNA were determined by measuring absorbance at 260 nm and the *A*_260_/*A*_280_ ratio using a microspectrophotometer (Nanophometer, Germany). RNA samples were stored at -80°C until further use.

### Ethics Statement

All procedures in the animal experiments were approved by the South China Agricultural University Experimental Animal Welfare Ethics Committee with a reference number of 2016-07.

### RNA Sequencing and Data Analysis

Eight total RNA samples were obtained to generate RNA libraries for each sample. After the samples were qualified, using mRNA Capture Beads Enriched eukaryote mRNA, mRNA was fragmented by heating. These short mRNA and random hexamers were used to generate the first cDNA and then the second cDNA was synthesized. The second cDNA was purified using VAHTSTM DNA Clean Beads (Vazyme, Nanjing, China) and then ligated to sequencing adapters. The fragments were amplified by using polymerase chain reaction (PCR) and purified using VAHTSTM DNA Clean Beads and then sequenced using an Illumina HiSeq (Vazyme, Nanjing, China). Raw sequence data were assessed, and sequences containing adaptor tags and those of low quality were excluded. Filtered reads were used for subsequent analysis, and the unique reads were used to identify differentially expressed genes (DEGs) with |log2Ratio|≥ 1 and *q*-value |FDR|≤ 0.05.

### Small RNA Sequencing and Data Analysis

Eight RNA libraries were generated with total RNA from samples. Total RNA was extracted and different fragments of RNAs by polyacrylamide gel electrophoresis (PAGE) were separated. Polyacrylamide electrophoresis gels were used to purify fragments that were 18–30 nt in length and 5′- and 3′-ends adaptors were ligated. The PCR products were generated after reverse-transcription (RT)-PCR and isolated using PAGE. Then, Illumina HiSeq 2000 (Vazyme, Nanjing, China) was used to sequence the purified cDNAs. After excluding reads with 3′- and 5′-primer contaminants or a poly(A) tails shorter than 18 nt, those with low-quality or those without the insert tag, were compared to clean reads in databases to annotate all known small RNA sequences. The unannotated sequences were searched against known miRNA precursors and mature miRNAs identified as known miRNAs. Differentially expressed (DE) miRNAs between the different samples were measured by |log2Ratio|≥ 1 and *q*-value |FDR|≤ 0.05.

### MiRNA–mRNA Integrative Genomic Analysis

To elucidate the interaction network of miRNA–mRNA with positive and negative correlations, we constructed an miRNA–mRNA regulatory network. The DE mRNAs and miRNAs were collected from the mRNA list and miRNA list. Then, we used miRanda^1^ and TargetScan^2^ to confirm the relationship between miRNA and mRNA. Finally, the relationship between miRNA and mRNA was verified.

### Functional Analysis

Depending on the miRNA–mRNA integrative genomic analysis, we used Gene Ontology (GO) to identify biological themes for each negatively correlated miRNA–mRNA pair. There are three ontologies in GO: molecular function, cellular component, and biological process. Negative miRNA–mRNA correlations were imported into the KEGG database^3^ for pathway analysis.

### Real-Time qPCR

cDNA was synthesized with oligo (dt) primer for mRNA, using PrimeScript^TM^ RT Master Mix (Takara, Otsu, Japan). qPCR was performed using the SYBR Premix Ex Taq^TM^ (Tli RNaseH Plus) (Takara, Otsu, Japan) on the LC480 Real-Time PCR System (Roche, Basel, Switzerland) in accordance with the manufacturer’s instructions (the primers are listed in Supplementary Tables 1, 2). cDNA was synthesized with A tail for miRNA, miRcute Plus miRNA Firsr-Strand cDNA Synthesis Kit (Tiangen, Beijing, China), and qPCR was performed using miRcute Plus miRNA qPCR Detection Kit (Tiangen, Beijing, China) on the LC480 Real-Time PCR System (Roche, Basel, Switzerland) in accordance with the manufacturer’s instructions. GAPDH was used as an endogenous control gene for mRNA and U6 was used for miRNA. We used the 2^-ΔΔC_T_^ method to analyze these data. qPCR in each reaction was performed in triplicate, and the data were expressed as the mean ± standard error (*n* = 3).

## Results

### Experiment with Dogs Infected with H5N1 Influenza Virus

Following infection with H5N1 influenza virus, clinical symptoms were observed, including cough, running nose, and an increased temperature. After infection with the virus, nasal swabs were collected from 1 to 14 dpi, and the peak temperature was 40.6°C at the second day after infection. The highest virus titer of a nasal swab was 10^4.56^. Virus replication in lung and trachea tissues was detected at 3 and 7 dpi, and the mean viral titers of 3 and 7 dpi were 10^5.6^ and 10^2.16^ TCID_50_/ml in lung tissues, respectively, whereas it was 10^1.3^ and 10^0.5^ TCID_50_/ml, respectively, in the trachea.

#### Overview of the Transcriptome and miRNAome

##### Gene libraries and miRNA libraries

Eight gene libraries as shown in Supplementary Data Sheet 1 were collected to identify mRNA differentiation of beagles when infected with H5N1 influenza virus. The beagles in the control group (KL3, KT3, KL7, and KT7) and experiment group (LUN3, TRA3, LUN7, and TRA7) were represented in the eight libraries. The original results of sequencing data and assembling results are shown in Supplementary Tables 3, 4. Among all of these reads, >90% of the clean reads were mapped to the canine reference genome.

Total RNA from the lung and trachea were used to build small RNA libraries to assess the characteristics of miRNAs in the lung and trachea after infection. After removing the contaminant and adaptor sequences and filtering low-quality tags, 10.5–12.4 million clean reads were collected in eight samples (as shown in Supplementary Table 5). Clean reads included rRNAs, tRNAs, snRNAs, and snoRNAs. After other RNAs were removed, 291, 290, 290, 283, 280, 287, 282, and 288 mature miRNAs were found in LUN3, TR3, LUN7, TR7, KL3, KT3, KL7, and KT7, respectively.

#### Differentially Expressed miRNAs and Analysis

**FIGURE 1:**
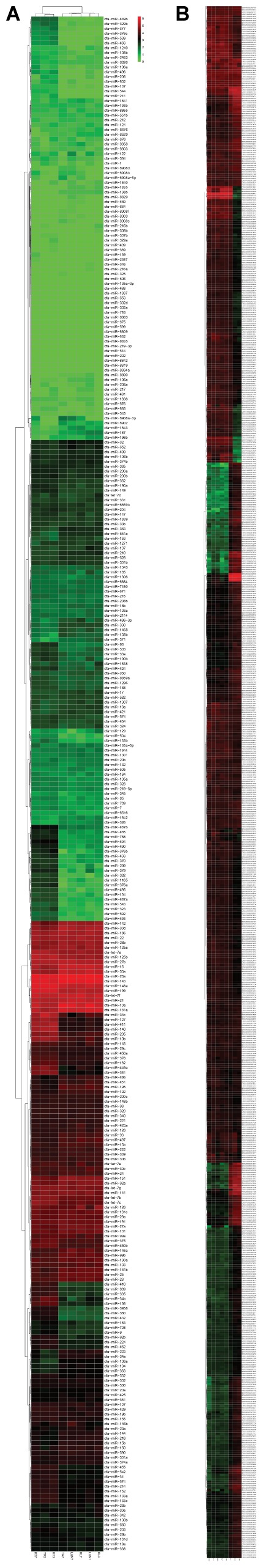
**(A,B)** Hierarchical clustering of DE miRNAs and DE mRNAs among eight RNA libraries. Heatmap of the number of DE miRNAs and DE mRNAs libraries for the DEGs between the infected groups and non-infected groups. MiRNA and mRNA heatmaps of all DE miRNAs and mRNAs are included.

**FIGURE 2:**
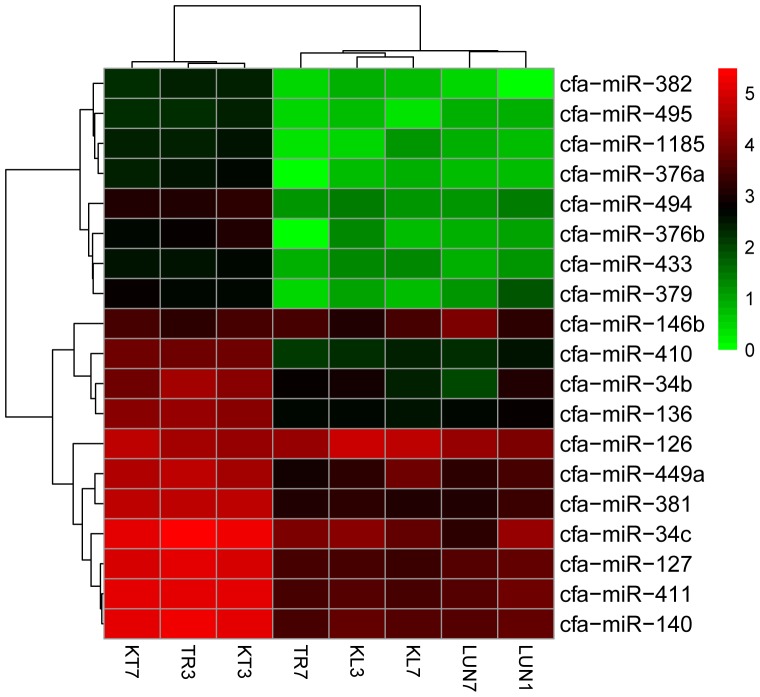
Clustering of the 19 up-regulated and down-regulated DE miRNAs for assigning dogs to infected groups and non-infected groups. The colors in the heat map represent the normalized expression values, with lower expression values indicated in shades of green and higher expression values in shades of red.

A total of 455 miRNAs were identified. The DE miRNAs were identified through |log2Ratio|≥ 1 and *q*-value |FDR|≤ 0.05 (as shown in **Figure 1A**). Compared with NI tissues, H5N1-infected tissues displayed differential expression of 19 mature miRNAs (as shown in **Figure 2**). Only miR-126 was DE on day 3 in lung tissue, miR-126 has been reported to restrict the replication of H5N1 in endothelial cells to ameliorate disease condition (Sant et al., 2017), probably implying that when infected with the virus, miR-126 may play an important role in restricting viral replication. MiR-34c, miR-146b, and miR-34b were DE on day 7 in lung tissue, playing anti-inflammatory (miR-146b) (Comer et al., 2014) and resistant apoptosis (miR-34) (Catuogno et al., 2012) roles at this stage. On the seventh day, the mi-34 family is predicted to protect the lung from undergoing apoptosis to restore lung function to normal. Fifteen DE miRNAs were identified on the seventh day in the tracheal tissue (as shown in Supplementary Table 6). At this stage, miRNAs may restore the state of the organism to normal; these included miR-379, which inhibited cell proliferation invasion (Li et al., 2017), anti-inflammatory-related miR-127 (Zhang et al., 2017), and apoptosis-related miR-410 (Palumbo et al., 2016; Deng H. et al., 2017).

**FIGURE 3:**
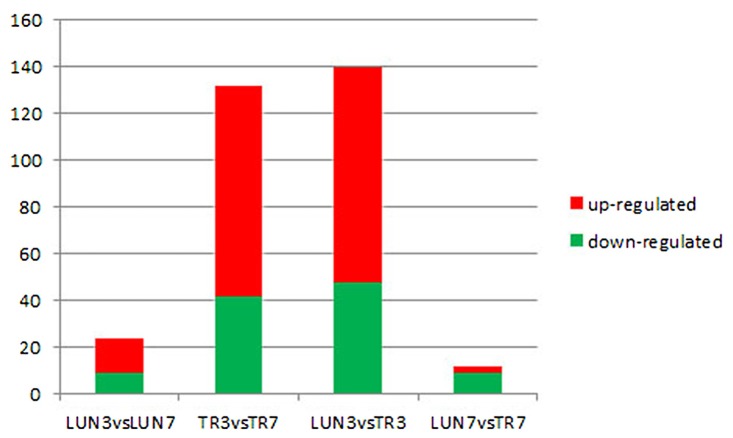
Four comparisons of different post-infection stages, miRNAs showing a differential expression pattern, with down-regulation indicated in green and up-regulation indicated in red.

**FIGURE 4:**
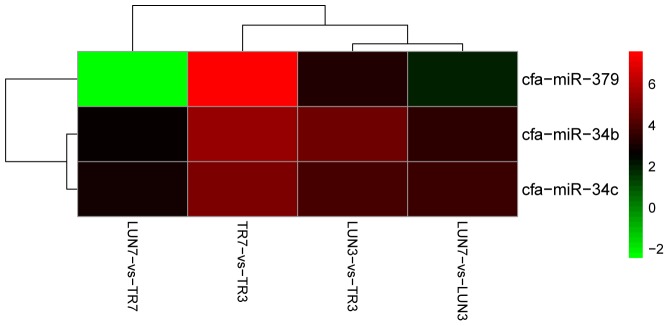
MiR-379, miR-34c, and miR-34b were DE at all different post-infection stages in four comparisons (LUN7 vs. TR7, TR7 vs. TR3, LUN3 vs. TR3, and LUN7 vs. LUN3).

To determine whether differential miRNA expression was related to differences in post-infection stages, four comparisons were made. Within the tissues, 24 and 132 different miRNAs were identified from lung and tracheal tissues at different times, respectively. Furthermore, 140 and 12 miRNAs were DE at 3 and 7 dpi, respectively (Supplementary Table 7). There were more up-regulated miRNAs (15 of 24 in the lung and 90 of 132 in the trachea) than down-regulated miRNAs in the lung and trachea (**Figure 3**). Furthermore, three miRNAs, miR-379, miR-34c, and miR-34b, were DE at different times (**Figure 4**). These results suggest that when infected with H5N1 influenza virus, miR-379 and miR-34 probably play significant roles in resisting viral invasion.

### Identification and Analysis of Differentially Expressed Genes

**FIGURE 5:**
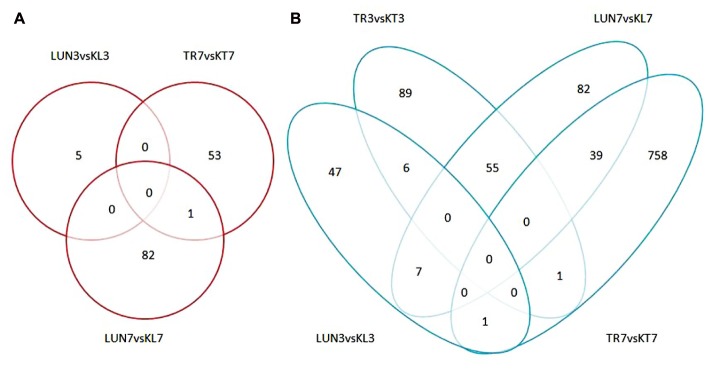
**(A)** Numbers of up-regulated DEGs in infected groups and non-infected groups. **(B)** Numbers of down-regulated DEGs in infected groups and non-infected groups. An FDR of 0.05 was used to classify genes as DE. The lung and tracheal tissues from beagles in the control group, named KL3 and KT3 on the third day and KL7 and KT7 on the seventh day, and the experimental group named LUN3 and TRA3 on the third day and LUN7 and TRA7 on the seventh day.

Compared with the control group, 1236 significant DEGs (**Figure 1B**) were identified. There were more down-regulated genes than up-regulated genes (**Figures 5A,B**). Among the down-regulated genes, 7 were found in the lung at different times, only 1 was found in the trachea at a different time, and 6 and 39 were found at 3 and 7 dpi, respectively; these included immune-related genes *IL2R2* (Schliemann et al., 2008), *CLEC4D* (Steichen et al., 2013), *IGFBP2* (Ambrosini-Spaltro et al., 2011), and *GPNMB* (Schwarzbich et al., 2011) and antiviral genes *FOSB* (Baumann et al., 2003) and *TNIP3* (Zhao et al., 2017).

### KEGG Pathway Enrichment Analysis of Differentially Expressed (DE) mRNAs

**FIGURE 6:**
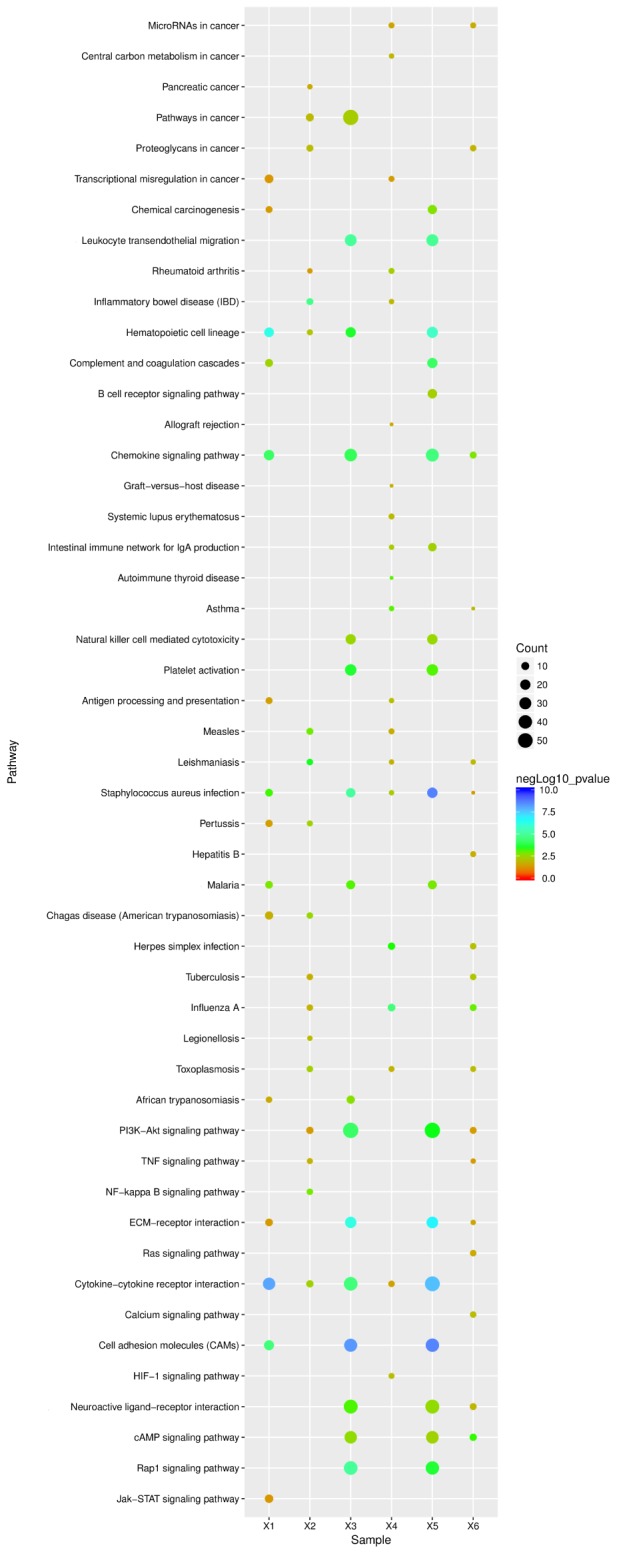
KEGG pathway enrichment analysis of DE unigenes of negative miRNA–mRNA and DE unigenes of RNA-seq. X1, 138 negative miRNA–mRNA interactions with 24 mature miRNAs and 201 validated mRNAs DE in the lung (15 pathways); X2, 916 DE unigenes of RNA-seq in lung tissue (18 pathways); X3, 134 negative miRNA–mRNA interactions with 97 mature miRNAs and 135 mRNAs were found in the trachea (16 pathways); X4, 2314 DE unigenes of RNA-seq in trachea tissue (20 pathways); X5, 65 mature miRNAs and 78 mRNA with 120 negative miRNA–mRNA interactions were identified under the infected state with different tissues (18 pathways); X6, 2411 DE unigenes of RNA-seq were identified under the infected state with different tissues (pathways). The ordinate represents the pathway name, the abscissa represents the sample name, the size of the dots indicates the number of DEGs in this pathway, and the point colors correspond to different negLog10 *p*-value ranges.

To explore the mechanisms underlying resistance to influenza virus infection in dogs, three groups were formed. We identified 919 and 2314 significant DEGs from lung and tracheal tissues, respectively; furthermore, DE genes were also identified under the infected state in the lung and tracheal tissues; 2411 DEGs were collected under the infected state in different tissues. These data were imported into KEGG databases. Significant (*p*-value < 0.05) pathways were identified (**Figure 6** X2, X4, and X6). The signaling pathway and immune related were the two most represented subunits among these groups. The chemokine signaling pathway and hematopoietic cell lineage belonged to the immune system, and all participated in the regulation of these groups. All were involved in the modulation of infected CIV signal pathways including cytokine–cytokine receptor interactions, the PI3K-Akt signaling pathway, cell adhesion molecules (CAMs), and ECM–receptor interactions. Apart from these, infectious diseases were another subclass pathway. Furthermore, influenza A was in the enriched pathway in all of the groups. These results suggested that genes in these pathways may play an important role in response to influenza virus.

### Integrative Genomic Analysis Associated with Negative miRNA–mRNA

To further investigate miRNA–mRNA regulatory information in dogs, transcriptome analyses were performed to identify genes that were co-expressed with miRNAs and miRNA targets were also predicted to identify genes that were bound to miRNA. A total of 268 and 251 miRNA–mRNA pairs with both positive and negative correlations, respectively, were identified in the lung and tracheal tissues, respectively; moreover, under the infected state, miRNA–mRNA pairs were also different between lung and tracheal tissues, and 255 miRNA–mRNA pairs were identified under the infected state in different tissues. However, miRNA generally negatively targeted genes. Therefore, when miRNAs are induced by the influenza virus, their target mRNAs are down-regulated and vice versa. There were 138 negative miRNA–mRNA interactions with 24 mature miRNAs and 201 validated mRNAs were DE in the lung. A total of 134 negative miRNA–mRNA interacted with 97 mature miRNAs and 135 mRNAs were found in the trachea. Moreover, 65 mature miRNAs and 78 mRNAs with 120 negative miRNA–mRNA interactions were identified under the infected state in different tissues. All target genes of the miRNAs were predicted using miRanda and TargetScan.

### Functional Annotation and Pathways Affected by Relevant Negative miRNA–mRNA Correlations in Beagles

To identify enriched functional terms of these predicted target genes and to further explore the significant negatively correlated miRNA–mRNA pairs, a GO analysis was carried out on these 105 mRNAs in the lung, 84 mRNAs in the trachea, and 78 mRNAs in different infected tissues, which were up-regulated and down-regulated. GO enrichment analysis was involved in biological processes, cellular components, and molecular functions.

**FIGURE 7:**
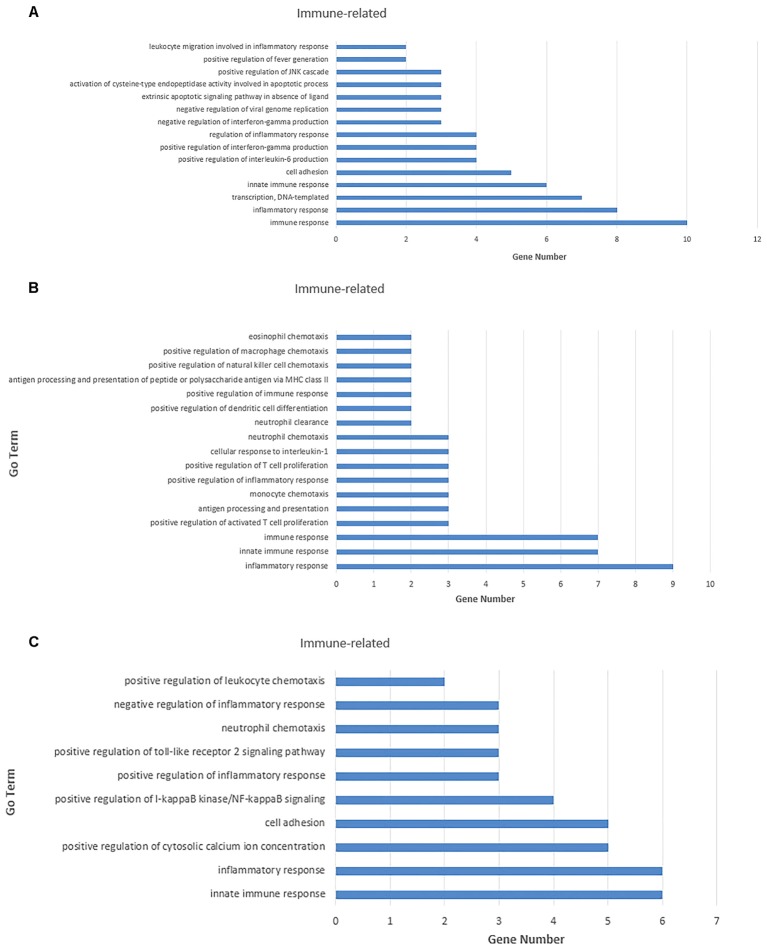
Gene ontology (GO) enrichment analysis for negatively correlated miRNA–mRNA. **(A)** Enriched immune-related GO terms of 109 target genes of repressed DE miRNAs in the lung tissue. **(B)** Enriched immune-related GO terms of 135 target genes of repressed DE miRNAs in the trachea tissue. **(C)** Enriched immune-related GO terms of 141 target genes of repressed DE miRNAs under the infected state with different tissues. The abscissa represents the sample name, and the numbers indicate related genes.

For the 109 mRNAs in the lung, 15 immune-related GO terms in biological process were significantly enriched (*p* < 0.05) (**Figure 7A**), and immune response was the highest fold enrichment (eightfold). Inflammatory responses, transcription, and positive regulation of fever generation were also identified. Furthermore, 17 immune-related GO terms in biological processes were significantly enriched (*p* < 0.05) in the trachea of 84 mRNAs (**Figure 7B**). Inflammatory responses and innate immune responses had the highest fold enrichment (ninefold). In addition, an infected state with different tissues was associated with 10 immune-related GO terms in biological processes (**Figure 7C**) including positive regulation of cytosolic calcium ion concentrations, positive regulation of I-kappaB kinase/NF-κB signaling, and positive regulation of the Toll-like receptor (TLR) 2 signaling pathway (*p* < 0.05). *In vivo*, different genes coordinate each other to perform biological functions. Pathway analysis helps to gain a better understanding of the biological function of genes.

Pathway enrichment analysis for 109 mRNAs of 138 negative miRNA–mRNA pairs in the lung, 84 mRNAs of 134 negative pairs in the trachea, and 78 mRNAs of 120 negative miRNA–mRNA pairs in different infected tissues. Further analysis found that among these significant (*p* < 0.05) pathways, signal- and immune-related pathways were also the most in all of these groups. In addition, infectious diseases were also another enrichment class of the pathways, and the influenza A pathway was also enriched in all groups (**Figure 6** X1, X3, and X5).

**FIGURE 8:**
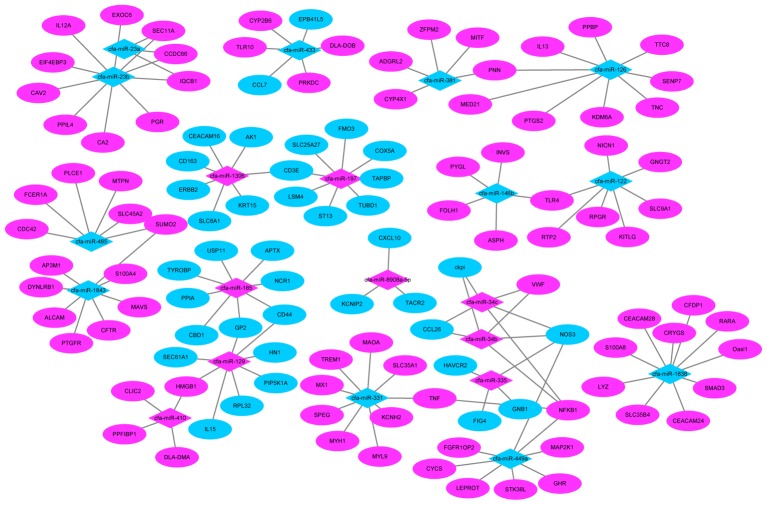
MiRNA–mRNA negative correlation network. Red indicates up-regulation and green indicates down-regulation.

We also found several genes played roles in multiple pathways. For example, TLR4 (miR-122) and nuclear factor kappa B subunit 1 (NFKB1) (miR-34c) were all involved in the influenza A pathway, as well as the TLR signaling pathway. While tumor necrosis factor (TNF) (miR-331) and nitric oxide synthase 3 (NOS3) (miR-335) participate in the PI3K-Akt signaling pathway and TNF signaling pathway (**Figure 8**).

#### qPCR Validation of DEGs

Seventeen DE mature miRNAs (cfa-miR-122, cfa-miR-129, cfa-miR-1838, cfa-miR-185, cfa-miR-23a, cfa-miR-331, cfa-miR-34c, cfa-miR-410, cfa-miR-433, cfa-miR-449a, cfa-miR-1843, cfa-miR-8908a-5p, cfa-miR-335, cfa-miR-34b, cfa-miR-485, cfa-miR-126, and cfa-miR-370) and 19 DEGs among the negative miRNA–mRNA interaction network were validated by qRT-PCR. The validation with qPCR was consistent with the sequencing results (as shown in **Tables 1, 2**).

**Table 1:** Relative miRNA expression of selected differentially expressed genes (DEGs) as revealed through miRNA-Seq and quantitative real-time PCR analyses.

miR_name	Illumina miRNA-seq	Regulation	Real-time PCR
	(log2 fold change)		(log2 fold change)
cfa-miR-122	-1.21676769	Down	-5.63666666666667^∗^
cfa-miR-129	3.53389948	Up	6.78333333333334^∗^
cfa-miR-1838	-1.07414525	Down	-6.54333333333334^∗^
cfa-miR-185	1.12327887	Up	6.42333333333334^∗^
cfa-miR-23a	-1.2160282	Down	-6.08^∗^
cfa-miR-331	1.06565908	Up	6.77666666666667^∗^
cfa-miR-34c	-2.725446079	Down	-9.88333666666666^∗^
cfa-miR-410	-5.370665561	Down	-3.22666666666667^∗^
cfa-miR-433	-5.630137818	Down	-4.88333333333334^∗^
cfa-miR-449a	-5.107389669	Down	-3.62666666666667^∗^
cfa-miR-1843	-1.29413691	Down	-5.89666666666667^∗^
cfa-miR-8908a-5p	2.78257022	Up	4.22666666666667^∗^
cfa-miR-335	4.08439398	Up	2.80666666666668^∗^
cfa-miR-34b	4.63988339	Up	1.66666666666667^∗^
cfa-miR-449a	4.19807289	Up	3.915^∗^
cfa-miR-485	4.9041897	Up	1.83333333333334^∗^
cfa-miR-126	1.26672194	Up	3.98^∗^
cfa-miR-370	4.9041897	Up	2.66666666666667^∗^

*^∗^Asterisk indicates statistical significance of differential gene expression with *p*-value < 0.05 (*t*-test).*

**Table 2:** Relative mRNA expression of 10 selected DEGs as revealed through mRNA-Seq and Quantitative real-time PCR.

Annotation	Accession	Illumina mRNA-seq	Regulation	Real-time PCR
		(log2 fold change)		(log2 fold change)
Toll-like receptor 4 (TLR4)	ENSCAFG00000003518	0.0576201	Up	0.480000000000004
Glycoprotein 2 (GP2)	ENSCAFG00000018023	-2.4609	Down	-2.53333333333333^∗^
Lysozyme (LYZ)	ENSCAFG00000000426	2.36132	Up	2.09^∗^
Interleukin 12A (IL12A)	ENSCAFG00000014200	-2.4609	Down	-0.973333333333333^∗^
Tumor necrosis factor (TNF)	ENSCAFG00000000517	0.0758078	Up	0.663333333333327
G-protein subunit gamma transducin 2 (GNGT2)	ENSCAFG00000016918	2.38011	Up	0.583333333333329
Interleukin 13 (IL13)	ENSCAFG00000000878	1.14698	Up	0.515000000000004
Nuclear factor kappa B subunit 1 (NF-κB1)	ENSCAFG00000010730	0.0836651	Up	1.97666666666666^∗^
Major histocompatibility complex, class II, DM alpha (DLA-DMA)	ENSCAFG00000000848	0.834172	Up	1.70500000000001^∗^
Major histocompatibility complex, class II, DO beta (DLA-DOB)	ENSCAFG00000000819	1.58298	Up	2.85666666666667^∗^
Nitric oxide synthase 3 (NOS3)	ENSCAFG00000004687	0.47985	Up	2.80666666666667^∗^
C–C motif chemokine ligand 5 (CCL5)	ENSCAFG00000018171	-0.952333	Down	-0.925000000000001^∗^
Interleukin 15 (IL15)	ENSCAFG00000003626	-1.19931	Down	-0.388333333333332
Cystic fibrosis transmembrane conductance regulator (CFTR)	ENSCAFG00000003429	0.887699	Up	0.418333333333333
Chemokine (C–X–C motif) ligand 10 (CXCL10)	ENSCAFG00000008584	-2.12935	Down	-0.9^∗^
Nitric oxide synthase 3 (NOS3)	ENSCAFG00000004687	-1.4553	Down	-0.694999999999997
Phospholipase C epsilon 1 (PLCE1)	ENSCAFG00000007985	-0.983665	Down	-0.326666666666664
Tenascin C (TNC)	ENSCAFG00000003426	-1.1546	Down	-1.02166666666667^∗^
Adrenoceptor alpha 1B (ADRA1B)	ENSCAFG00000017281	-1.5188	Down	-0.594999999999999

*^∗^Asterisk indicates statistical significance of differential gene expression with *p*-value < 0.05 (*t*-test).*

## Discussion

H5N1 infections are characterized with a high-fatality rate; hence, in this study, we choose beagles as the animal model of H5N1 infection. We investigated the effect of H5N1 virus infection on miRNA and mRNA, and analyzed the interaction between miRNA and mRNA. Dysregulation of 24 miRNAs and 204 mRNAs with 138 negative miRNA–mRNA pairs was observed in the lung tissue, and dysregulation of 97 miRNAs and 135 mRNAs with 134 negative miRNA–mRNA pairs was observed in the tracheal tissue; moreover, under the infected state, between lung and trachea, miRNAs and mRNAs were also different expressed: 97 miRNAs and 141 mRNAs with 120 negative miRNA–mRNA pairs were found between different tissues. Target mRNA gene functions were analyzed with GO and the KEGG database. Several pathways were activated by relevant target genes of DE miRNAs with a negative miRNA–mRNA correlation.

Infection with H5N1 influenza virus caused differential expression of mRNA in lung and trachea tissues. Comparisons among these infected groups revealed 65 similar up-regulated genes and 7 similar down-regulated genes. These up-regulated genes included antiviral genes, such as cell surface receptor (*CD59*), calcium ion binding gene (*EFHC1* and *CAPS2*), and protein coding genes (*RIBC2, CFAP157, DRC7, CFAP45, CFAP65*, and *CCDC33*). *CD59* (Zhang et al., 2010) can inhibit local inflammatory reactions caused by the influenza virus. *EFHC1* affects cellular apoptosis (Wang et al., 2002; Katano et al., 2012). *CFAP157* (Benos et al., 2012), *DRC7* (Yang et al., 2011), and *CFAP45* (Li et al., 1999) are involved in the regulation of flagella and cilia motility for viral invasion. Of the down-regulated genes, *CD5L* combined with any modified low-density lipoprotein (LDL) or other polyanionic ligand and delivered the ligand into the cell via receptor-mediated endocytosis to regulate innate immunity (Peiser et al., 2002). SLC11A1 is a natural resistance-associated macrophage protein (NRAMP1), which is expressed only in immune-related cells (Govoni and Gros, 1998). As a divalent transition metal transporter, it was also involved in iron metabolism and assisted with host resistance to certain pathogens. In conclusion, when infected by H5N1 CIV, immune-related genes and antiviral genes are activated to resist viral infection.

Differential miRNA expression in the lung and trachea revealed that the regulatory mechanism of miRNA on host responses when infected with CIV and that between lung and trachea is different. At different post-infection stages, there were more miRNAs expressed at 3 than 7 dpi. Immunogenicity was enhanced by miR-155 (Izzard et al., 2017), miR-375 (Lin et al., 2017), miR-155, miR-146a (Zhou et al., 2017), and miR-136 (Zhao et al., 2015). Furthermore, miR-146a (Terrier et al., 2013) plays an antiviral role. Moreover, miR-29 activates cyclooxygenase and lambda-1 IFN to resist viral infection (Fang et al., 2011). As a cytolytic virus, influenza A virus induces apoptosis, which results in organ and cellular dysfunction. MiR-15b and miR-451 regulate a series of pro-inflammatory cytokine responses (Chan et al., 2011; Rosenberger et al., 2012). Moreover, miR-29c inhibits *BCL2L2* expression to regulate apoptosis induced by influenza A virus (Guan et al., 2012). These data reveal that viral infections typically induce miRNAs that regulate cytokine production and the anti-viral immune response.

The present study reveals novel findings regarding H5N1 influenza virus-infected dogs and lung and tracheal tissue profiling, since these have not been previously performed. MiRNAs and their target genes have multiple relationships (Perez et al., 2009; Fan and Wang, 2016; Nakamura et al., 2016). Dysregulated miRNAs may serve as a diagnostic and prognostic biomarker (Okkenhaug and Vanhaesebroeck, 2003; Hale et al., 2010; Haneklaus et al., 2013; Ingle et al., 2015).

Influenza, caused by influenza virus, is an acute febrile contagious respiratory infectious disease. The innate immune system recognizes invading viruses (Hale et al., 2010; Iwasaki and Pillai, 2014) through multiple mechanisms. The non-structural NS1 inhibits type I IFN production (Haye et al., 2009; Pekosz et al., 2012) by inactivating transcription factors (MacEwan, 2002; Wang et al., 2017) such as IRF3 (Wang et al., 2017), AP1, and NF-κB (miR-34) (Taganov et al., 2006). Pattern recognition receptors (PRRs) recognize viral RNA (Thompson et al., 2011; Okamoto et al., 2017) to activate a specialized immune response at mucosal surfaces to combat viral invasion. TLR4 (miR-146) has been reported to attenuate influenza virus infection with TLR4 antagonists, which may be a novel therapeutic approach to combat infection (Shirey et al., 2013). MiR-146a regulates TRAF6 when infected with H3N2 virus (Deng Y. et al., 2017). Moreover, TRAF6 and MEKK1 are critical genes that IPS-1 activates NF-κB and induces IFNs (Yoshida et al., 2008). In addition, miR-144 targets TRAF4-IRF7 through NF-κB to attenuate the host response to influenza virus (Lopez et al., 2017). Furthermore, miR-34 targets pro-apoptosis Bax through downregulation (Fan and Wang, 2016) during an influenza virus infection. Despite the paucity in the number of studies on dogs, it is important to refer to previous studies. Briefly, NF-κB and TLR4 with their target miR-34c and miR-146 through IPS-1 and TRAF family may have important roles in regulating the innate immune system during an H5N1 CIV infection.

Toll-like receptors activate IFNs and lead to antiviral responses through cytokine–cytokine receptor interactions. As chemokines (Zlotnik and Yoshie, 2000), ccl5 (miR-335) and cxcl10 (miR-8908a-5p) activate their receptors once activated, different downstream pathways are activated and may produce a series of immune responses. In addition, P13K-Akt signaling pathway regulates transcription, growth, proliferation, translation, and survival of fundamental cellular functions (Koyasu, 2003; Okkenhaug and Vanhaesebroeck, 2003; Richardson et al., 2004; Duronio, 2008; Hers et al., 2011). When cells are infected with the influenza A virus, viral NS1 binds to and activates P13K, inducing the beta-IFN and the apoptotic responses (Ehrhardt et al., 2007; Ehrhardt and Ludwig, 2009). In our findings, up-regulated GNGT2 and down-regulated miR-122 activate the P13K-Akt pathway during an influenza virus infection. Despite the paucity in the number of studies on canine microRNA and mRNA, it is important to refer to previous studies to explain the function of microRNAs in dogs and is of great significance in studies on dogs.

Although the expression levels and functions of miRNAs in the lung and trachea have been studied, the effects of beagles infected with influenza virus on mRNA and miRNA expression and influenza virus-related pathways have not been investigated. To our knowledge, this is the first study to assess the effect of beagles on miRNA–mRNA expression when infected with H5N1 influenza virus. Despite the low chance of H5N1 infection in domestic animals, direct contact of H5N1-infected dogs with humans may occur. Our results provide information for understanding the mechanisms of viral pathogenesis in dogs.

## Author Contributions

CF contributed to the data analysis and the writing of the manuscript. JL contributed to the drafting of the manuscript. SY and ZY contributed to the data collection and the laboratory work. CF and JL contributed to the animal experiment. SL contributed to the conception of the idea and design. All authors read and approved the manuscript.

## Conflict of Interest Statement

The authors declare that the research was conducted in the absence of any commercial or financial relationships that could be construed as a potential conflict of interest.

## Footnotes

1 http://34.236.212.39/microrna/home.do

2 http://www.targetscan.org

3 http://www.genome.jp/kegg/
