# Supplementary material for: Integrated Lung and Tracheal mRNA-Seq and miRNA-Seq Analysis of Dogs with an Avian-Like H5N1 Canine Influenza Virus Infection
Source: Front Microbiol. 2018 Mar 5;9:303. doi: 10.3389/fmicb.2018.00303 (PMC5844969; doi:10.3389/fmicb.2018.00303)
Supplement: Supplementary file 2 [file Data_Sheet_2.docx]

Supplementary Material

**Integrated analysis of mRNA-seq and miRNA-seq in the lung and trachea of dogs in response to avian-like H5N1 canine influenza virus**

***Cheng Fu^1,2^, Jie Luo^1,2^, Shaotang Ye^1,2^, Ziguo Yuan^1，2^, Shoujun Li^1,2^****

*** Correspondence: Shoujun Li. Email:** [**shoujunli@scau.edu.cn**](mailto:shoujunli@scau.edu.cn)**.**

**1 Supplementary Figures and Tables**

**Table 1 mRNA Primers used in this study.**

| Name of genes | Forward primer (5′-3′) | Reverse primer (5′-3′) |
| --- | --- | --- |
| toll like receptor 4(TLR4) | GCCTAAACCACCTCTCCATCTTGA | GCCACCAGCGTCTTTAAACTTGAT |
| glycoprotein 2(GP2) | AAGTGTGTGACAAGGACAAGCATG | CATCTGTCTGGCATCGGTACATTG |
| lysozyme(LYZ) | GAGGGTTGTCAGTGATCCAAATGG | ACACTCCACAATTCCGAACATACTG |
| interleukin 12A(IL12A) | TAACCATGAATGAGAGTTGCCTGG | CAAGTCCTCATAGATGCTGCTAAGG |
| tumor necrosis factor(TNF) | CAACTGGAGAAGGGTGATCGAC | ACAGGGCAATGATTCCAAAGTACA |
| nuclear factor kappa B subunit 1(NFKB1) | GAGGGTTGTCAGTGATCCAAATGG | ACACTCCACAATTCCGAACATACTG |
| cystic fibrosis transmembrane conductance regulator(CFTR) | ATTGGAATGCAGATCAGAATAGCT | CTTCATCGAATTTGTTCAGGTTGT |
| chemokine (C-X-C motif) ligand 10(CXCL10) | ATTGAGATGATTCCTGCAAGTCCA | AAACTCTTGATGGTCTTAGATTCTGGA |
| interleukin 13(IL13) | GCATTACAGTGGAGGCAGATATGT | CCCAGCACAAACAAAGACACTTG |
| nitric oxide synthase 3(NOS3) | GAAGAGTCCAGTGAACAGCAGGAG | GTTCCAGTACCTCCAGCAGTGTG |
| phospholipase C epsilon 1(PLCE1) | GGACGGAAAGCACACCAGTTTC | CTTCCTCCATGACAGAATTGCACG |
| tenascin C(TNC) | GTCTGTGGAAGTGGAGTGGGAT | TTTGGTGATCTCTCCCTCATCTTCTT |
| adrenoceptor alpha 1B(ADRA1B) | CTACCCTTCTTCATCGCTCTACCG | CTGTTGAAGTAGCCCAGCCAGA |
| major histocompatibility complex, class II, DM alpha(DLA-DMA) | CATGAGATTGACAACTACACGGCA | TGAGGACTAAGCCAACAATGATGC |
| major histocompatibility complex, class II, DO beta(DLA-DOB) | CATGGATGCTGGCTCTGTTAGTGA | ACAAACTGCACCTTCTCTGTTCCA |
| C-C motif chemokine ligand 5(CCL5) | CAGAAGAAATGGGTGCGGGAGTA | CAAGAAGCAGTAGGAAAGTTTGCATG |
| interleukin 15(IL15) | CAGGACGTGATACTTGATTTGGAAA | CGGTTACTTTGCAACTGGGATG |
| G protein subunit gamma transducin 2(GNGT2) | CTCTCCTCTACTCCACCTGCT | GGCTCTTTGAATCCCACCAC |

**Table2 miRNA Primers used in this study.**

| Name | Forward Primer (5′-3′) | Reverse primer (5′-3′) |
| --- | --- | --- |
| cfa-miR-122 | CGTGGAGTGTGACAATGGTGTTG | TGCTGTCACGATACGCTACGTAACG |
| cfa-miR-129 | CTTTTTGCGGTCTGGGCTTG | TGCTGTCACGATACGCTACGTAACG |
| cfa-miR-1838 | CACCAGCTGGCGTTCCCT | TGCTGTCACGATACGCTACGTAACG |
| cfa-miR-185 | TGGAGAGAAAGGCAGTTCCTGAG | TGCTGTCACGATACGCTACGTAACG |
| cfa-miR-23a | ATCACATTGCCAGGGATTTGGCC | TGCTGTCACGATACGCTACGTAACG |
| cfa-miR-331 | GCCCCTGGGCCTATCCTAGAAG | TGCTGTCACGATACGCTACGTAACG |
| cfa-miR-34c | AGGCAGTGTAGTTAGCTGATTGCVG | TGCTGTCACGATACGCTACGTAACG |
| cfa-miR-1843 | ACTGGAGGTCTCTGTCTGGCTT | TGCTGTCACGATACGCTACGTAACG |
| cfa-miR-8908a-5p | TGCTGAGGAAGGTGCTCATTGCATT | TGCTGTCACGATACGCTACGTAACG |
| cfa-miR-34b | AGGCAGTGTAATTAGCTGATTGCGC | TGCTGTCACGATACGCTACGTAACG |
| cfa-miR-126 | CATTATTACTTTTGGTACGCGGCGC | TGCTGTCACGATACGCTACGTAACG |
| cfa-miR-335 | TCAAGAGCAATAACGAAAAATGTCCGGC | TGCTGTCACGATACGCTACGTAACG |
| cfa-miR-449a | TGGCAGTGTATTGTTAGCTGGTCCG | TGCTGTCACGATACGCTACGTAACG |
| cfa-miR-485 | AGAGGCTGGCCGTGATGAATT | TGCTGTCACGATACGCTACGTAACG |
| cfa-miR-370 | CCTGCTGGGGTGGAACCTG | TGCTGTCACGATACGCTACGTAACG |
| cfa-miR-410 | AATATAACACAGATGGCCTGTCCGGG | TGCTGTCACGATACGCTACGTAACG |
| cfa-miR-433 | ATCATGATGGGCTCCTCGGTGT | TGCTGTCACGATACGCTACGTAACG |
| U6 | ACTAAAATTGGAACGATACAGAGA | AAAGATGGAACGCTTCACG |

**Table3 Comparison of sequence data alignment**

| Samples | Total clean reads | Total mapped reads | PE mapped reads | Left mapped reads | Right mapped reads |
| --- | --- | --- | --- | --- | --- |
| KL3 | 89481504 | 81586525(91.2%) | 76601996(84.2%) | 41141677(92.0%) | 40444848(90.4%) |
| KT3 | 72724428 | 65786518(90.5%) | 61463834(83.0%) | 33245922(91.4%) | 32540596(89.5%) |
| KL7 | 77721136 | 70477555(90.7%) | 66316594(84.0%) | 35532961(91.4%) | 34944594(89.9%) |
| KT7 | 77458250 | 69756476(90.1%) | 65414932(83.1%) | 35217442(90.9%) | 34539034(89.2%) |
| LUN3 | 64430434 | 58814030(91.3%) | 55340016(84.8%) | 29631800(92.0%) | 29182230(90.6%) |
| TR3 | 62776628 | 57293851(91.3%) | 53934792(84.6%) | 28891209(92.0%) | 28402642(90.5%) |
| LUN7 | 58373358 | 53285447(91.3%) | 50106760(84.5%) | 26848628(92.0%) | 26436819(90.6%) |
| TR7 | 67103036 | 61320294(91.4%) | 57680750(84.8%) | 30876582(92.0%) | 30443712(90.7% |

**Table4 Original sequencing data**

| Samples | Read_length | Raw_reads | Clean_reads | Clean_bases | Raw_GC | Clean_GC | Raw_Q20 | Clean_Q20 | Raw_Q30 | Clean_Q30 | Adapter |
| --- | --- | --- | --- | --- | --- | --- | --- | --- | --- | --- | --- |
| KL3 | 150 bp | 89687726 | 89481504 | 13422225600 | 49.43% | 49.42% | 98.21% | 98.22% | 95.82% | 95.84% | 0.23% |
| KT3 | 150 bp | 72942440 | 72724428 | 10908664200 | 50.43% | 50.42% | 98.10% | 98.11% | 95.66% | 95.67% | 0.30% |
| KL7 | 150 bp | 78046156 | 77721136 | 11658170400 | 48.96% | 48.95% | 98.29% | 98.31% | 96.03% | 96.06% | 0.42% |
| KT7 | 150 bp | 77782356 | 77458250 | 11618737500 | 49.70% | 49.70% | 98.16% | 98.19% | 95.78% | 95.81% | 0.42% |
| LUN3 | 150 bp | 64622938 | 64430434 | 9664565100 | 49.71% | 49.71% | 98.28% | 98.30% | 95.97% | 95.98% | 0.30% |
| TR3 | 150 bp | 63044626 | 62776628 | 9416494200 | 49.64% | 49.64% | 98.28% | 98.29% | 95.97% | 95.98% | 0.42% |
| LUN7 | 150 bp | 58682692 | 58373358 | 8756003700 | 49.86% | 49.86% | 98.30% | 98.32% | 96.01% | 96.03% | 0.52% |
| TR7 | 150 bp | 67372822 | 67103036 | 10065455400 | 50.02% | 50.02% | 98.35% | 98.37% | 96.09% | 96.11% | 0.40% |

**Table 5 miRNA data quality**

|  | LUN3 | TR3 | KL3 | KT3 | LUN7 | TR7 | KL7 | KT7 |
| --- | --- | --- | --- | --- | --- | --- | --- | --- |
| total reads | 12462420 | 12656636 | 11515685 | 12791239 | 11586552 | 12614795 | 10812292 | 12892162 |
| high quality | 12430330 | 12626837 | 11488591 | 12760366 | 11559204 | 12584019 | 10786788 | 12877468 |
| 3' adapter null | 106214 | 10681 | 110867 | 119663 | 108072 | 130259 | 155016 | 94915 |
| insert null | 987 | 3396 | 3122 | 5816 | 1499 | 0.01 | 2660 | 2809 |
| 5' adapter contaminants | 1044 | 6457 | 7094 | 10657 | 1777 0.02 | 1192 | 2546 | 5489 |
| small than 18nt | 78173 | 573745 | 792466 | 466212 | 185551 | 124997 | 176038 | 288632 |
| polyA | 104 | 434 | 447 | 536 | 135 | 154 | 209 | 491 |
| clean reads | 12243808 | 11935994 | 10574595 | 12157482 | 11262170 | 12326691 | 10450319 | 12485132 |

**Table 6. List of differentially expressed miRNAs compared to non-infected groups**

| LUN7 vs KL7 | | | | | | | |
| --- | --- | --- | --- | --- | --- | --- | --- |
| miR_name | KL7-ReadsMean | LUN7-ReadsMean | KL7-baseMean | LUN7-baseMean | log2Ratio(LUN7/KL7) | P-value | FDR |
| cfa-miR-34c | 10158.5 | 1503 | 10012.57 | 1513.92571 | -2.725446079 | 2.69E-06 | 0.000402189 |
| cfa-miR-146b | 2318.5 | 12426 | 2338.5148 | 12516.3279 | 2.420146891 | 4.67E-07 | 0.000139717 |
| cfa-miR-34b | 507 | 99 | 498.55663 | 99.7196574 | -2.321807563 | 4.50E-05 | 0.004487629 |
| LUN3 vs KL3 | | | | | | | |
| miR_name | KL3-ReadsMean | LUN3-ReadsMean | KL3-baseMean | LUN3-baseMean | log2Ratio(LUN3/KL3) | P-value | FDR |
| cfa-miR-126 | 68199 | 11536 | 69301.001 | 11093.1546 | -2.643206508 | 1.13E-06 | 0.000346753 |
| TR7 vs KT7 | | | | | | | |
| miR_name | KT7-ReadsMean | TR7-ReadsMean | KT7-baseMean | TR7-baseMean | log2Ratio(TR7/KT7) | P-value | FDR |
| cfa-miR-449a | 34201 | 846 | 32117.195 | 931.665735 | -5.107389669 | 0.0001536 | 0.005170124 |
| cfa-miR-411 | 143188 | 3530 | 134576.71 | 3887.44686 | -5.113461957 | 0.0002296 | 0.005796417 |
| cfa-miR-140 | 144772.5 | 2898 | 136085.66 | 3191.45071 | -5.414158921 | 0.0001227 | 0.005170124 |
| cfa-miR-127 | 97259.5 | 2886 | 91404.821 | 3178.23559 | -4.845972282 | 0.000347 | 0.007009726 |
| cfa-miR-381 | 51989 | 1212 | 48868.113 | 1334.7268 | -5.194277032 | 0.000137 | 0.005170124 |
| cfa-miR-136 | 16163 | 427 | 15190.76 | 470.237906 | -5.013659357 | 0.0001875 | 0.005680168 |
| cfa-miR-410 | 7417.5 | 153 | 6971.2815 | 168.492739 | -5.370665561 | 0.0001009 | 0.005097014 |
| cfa-miR-433 | 406 | 7 | 381.79318 | 7.70881814 | -5.630137818 | 0.0003207 | 0.007009726 |
| cfa-miR-379 | 558.5 | 3 | 524.64483 | 3.3037792 | -7.311079997 | 3.05E-05 | 0.003082035 |
| cfa-miR-494 | 1528.5 | 16 | 1437.1983 | 17.6201557 | -6.349888688 | 6.52E-05 | 0.00419711 |
| cfa-miR-376b | 745 | 1 | 700.96101 | 1.10125973 | -9.314035615 | 3.57E-06 | 0.000541414 |
| cfa-miR-495 | 193.5 | 3 | 181.90579 | 3.3037792 | -5.782930343 | 0.0003386 | 0.007009726 |
| cfa-miR-1185 | 278.5 | 2 | 261.8194 | 2.20251947 | -6.893273417 | 6.93E-05 | 0.00419711 |
| cfa-miR-382 | 231 | 3 | 217.15138 | 3.3037792 | -6.038440045 | 0.0002131 | 0.005796417 |
| cfa-miR-376a | 392 | 0 | 368.6941 | 0.001 | -18.4920648 | 3.56E-06 | 0.000541414 |

**Table 7. List of differentially expressed miRNAs at different developmental stages**

| LUN7 LUN3 | | |
| --- | --- | --- |
| miR_name | fold-change(log2 LUN7/LUN3) | p-value |
| cfa-miR-107 | -1.05911681 | 7.50E-213 |
| cfa-miR-122 | -1.21676769 | 0.00985005 |
| cfa-miR-124 | 1.89029608 | 0.03130669 |
| cfa-miR-129 | 3.53389948 | 7.45E-06 |
| cfa-miR-1306 | 1.47517483 | 1.64E-05 |
| cfa-miR-146b | -3.08880559 | 0 |
| cfa-miR-1838 | -1.07414525 | 6.97E-05 |
| cfa-miR-185 | 1.12327887 | 2.70E-09 |
| cfa-miR-187 | 1.66784378 | 4.14E-05 |
| cfa-miR-190b | 1.29986793 | 3.62E-11 |
| cfa-miR-196b | 1.47520469 | 0.00874519 |
| cfa-miR-197 | 1.6169834 | 1.83E-54 |
| cfa-miR-23a | -1.2160282 | 4.02E-212 |
| cfa-miR-23b | -1.60752801 | 1.86E-169 |
| cfa-miR-299 | 1.89022765 | 0.00028831 |
| cfa-miR-331 | 1.06565908 | 6.64E-12 |
| cfa-miR-34b | 3.41821295 | 8.51E-209 |
| cfa-miR-34c | 3.76847663 | 0 |
| cfa-miR-379 | 1.96057702 | 1.44E-07 |
| cfa-miR-451 | 1.14508054 | 0 |
| cfa-miR-6516 | -1.1686321 | 0.0183051 |
| cfa-miR-652 | -1.26851334 | 5.40E-60 |
| cfa-miR-8908a-3p | -9.06441943 | 5.31E-16 |
| cfa-miR-92b | 1.69504464 | 3.36E-70 |
| LUN7 TR7 | | |
| miR_name | fold-change(log2 TR7/LUN7) | p-value |
| cfa-miR-122 | -8.28683495 | 1.04E-09 |
| cfa-miR-146b | -2.21347261 | 0 |
| cfa-miR-1843 | -1.29413691 | 0.03042898 |
| cfa-miR-34b | 2.70387882 | 3.07E-102 |
| cfa-miR-34c | 2.86959609 | 0 |
| cfa-miR-376b | -3.2945938 | 0.00782144 |
| cfa-miR-379 | -2.44621312 | 0.00244804 |
| cfa-miR-449a | -1.24825968 | 3.27E-104 |
| cfa-miR-485 | -1.01721566 | 0.03082638 |
| cfa-miR-505 | -1.27617746 | 0.00126782 |
| cfa-miR-652 | -1.03297362 | 7.59E-44 |
| cfa-miR-8908a-5p | 2.78257022 | 0.00238194 |
| LUN3 TR3 | | |
| miR_name | fold-change(log2 TR3/LUN3) | p-value |
| cfa-let-7b | 1.02929651 | 0 |
| cfa-let-7c | 1.73452853 | 0 |
| cfa-miR-105a | 1.1817753 | 0.00120375 |
| cfa-miR-107 | 1.22585157 | 8.89E-298 |
| cfa-miR-10a | -2.24352063 | 0 |
| cfa-miR-10b | 2.71020972 | 0 |
| cfa-miR-1185 | 5.9359906 | 1.98E-81 |
| cfa-miR-126 | 1.26672194 | 0 |
| cfa-miR-127 | 4.64351747 | 0 |
| cfa-miR-1296 | 1.01015137 | 8.09E-08 |
| cfa-miR-133a | 1.61578746 | 0 |
| cfa-miR-133b | 3.07525264 | 1.62E-15 |
| cfa-miR-133c | 1.61578746 | 0 |
| cfa-miR-134 | 3.6729213 | 1.51E-41 |
| cfa-miR-135b | -1.75757846 | 3.47E-10 |
| cfa-miR-136 | 4.8952512 | 0 |
| cfa-miR-138a | -2.86129641 | 1.02E-267 |
| cfa-miR-140 | 4.94576073 | 0 |
| cfa-miR-142 | -1.95246555 | 0 |
| cfa-miR-144 | -1.08863771 | 6.94E-150 |
| cfa-miR-1468 | -1.48202985 | 8.73E-12 |
| cfa-miR-146a | -1.66335616 | 0 |
| cfa-miR-147 | 2.0830908 | 2.19E-54 |
| cfa-miR-148a | 1.25637031 | 0 |
| cfa-miR-149 | 1.25531381 | 6.57E-29 |
| cfa-miR-150 | -1.61086334 | 6.41E-226 |
| cfa-miR-151 | -1.03511947 | 0 |
| cfa-miR-152 | 1.24846439 | 2.65E-295 |
| cfa-miR-153 | -1.11916436 | 1.04E-12 |
| cfa-miR-155 | 1.0608081 | 9.86E-197 |
| cfa-miR-15b | -1.45193826 | 1.98E-247 |
| cfa-miR-16 | -1.718206 | 0 |
| cfa-miR-181a | -2.25692693 | 0 |
| cfa-miR-181b | -1.62575479 | 0 |
| cfa-miR-182 | 2.18184749 | 0 |
| cfa-miR-183 | 1.87932502 | 2.76E-157 |
| cfa-miR-1838 | 1.50600839 | 2.17E-09 |
| cfa-miR-1843 | -2.43549717 | 0.01298729 |
| cfa-miR-185 | -1.08374506 | 9.92E-09 |
| cfa-miR-187 | -3.85071533 | 1.06E-10 |
| cfa-miR-188 | 1.0744647 | 5.28E-09 |
| cfa-miR-18a | -1.47040925 | 9.27E-18 |
| cfa-miR-190a | 1.21941059 | 5.84E-28 |
| cfa-miR-190b | 1.33536778 | 5.69E-27 |
| cfa-miR-192 | -1.54494667 | 0 |
| cfa-miR-194 | -1.7768383 | 5.80E-152 |
| cfa-miR-195 | -2.47941455 | 0 |
| cfa-miR-196b | -3.43549717 | 1.81E-05 |
| cfa-miR-197 | -1.97230547 | 6.83E-70 |
| cfa-miR-199 | 1.77541683 | 0 |
| cfa-miR-203 | 1.33544382 | 7.81E-108 |
| cfa-miR-205 | 2.26661551 | 0 |
| cfa-miR-206 | 2.65178061 | 0.00065582 |
| cfa-miR-214 | 1.20456426 | 5.46E-209 |
| cfa-miR-218 | -1.46573375 | 9.90E-267 |
| cfa-miR-223 | -3.85783042 | 0 |
| cfa-miR-224 | 2.53155501 | 0 |
| cfa-miR-25 | -1.25971045 | 0 |
| cfa-miR-26a | -1.1839388 | 0 |
| cfa-miR-299 | 3.45765415 | 5.07E-67 |
| cfa-miR-29b | 1.35659301 | 1.01E-96 |
| cfa-miR-29c | 1.37612719 | 0 |
| cfa-miR-30a | -1.91301837 | 0 |
| cfa-miR-30c | -1.02384154 | 0 |
| cfa-miR-30d | -1.96919985 | 0 |
| cfa-miR-31 | 2.22691397 | 0 |
| cfa-miR-32 | -1.05212382 | 3.78E-31 |
| cfa-miR-323 | 5.68327641 | 6.01E-41 |
| cfa-miR-326 | -1.68080487 | 0.00092086 |
| cfa-miR-329b | 5.43493885 | 4.57E-23 |
| cfa-miR-330 | -2.15691131 | 1.87E-21 |
| cfa-miR-335 | 4.08439398 | 0 |
| cfa-miR-33a | 2.07753351 | 9.67E-48 |
| cfa-miR-33b | 1.1084247 | 3.50E-16 |
| cfa-miR-34a | -2.47834595 | 0 |
| cfa-miR-34b | 4.63988339 | 0 |
| cfa-miR-34c | 3.93621352 | 0 |
| cfa-miR-363 | -1.84825954 | 3.38E-160 |
| cfa-miR-370 | 4.3192532 | 1.06E-76 |
| cfa-miR-371 | -3.5511138 | 1.40E-20 |
| cfa-miR-374a | -1.22269515 | 5.52E-256 |
| cfa-miR-374b | -1.25889616 | 2.12E-44 |
| cfa-miR-375 | 1.44321186 | 0 |
| cfa-miR-376a | 6.19815231 | 7.25E-99 |
| cfa-miR-376b | 6.06334108 | 1.14E-177 |
| cfa-miR-376c | 4.95617532 | 1.30E-08 |
| cfa-miR-377 | 5.23631496 | 1.76E-10 |
| cfa-miR-378 | 1.55605808 | 0 |
| cfa-miR-379 | 3.17766807 | 1.10E-99 |
| cfa-miR-380 | 4.55281022 | 0 |
| cfa-miR-381 | 4.72281146 | 0 |
| cfa-miR-382 | 11.31341593 | 1.07E-72 |
| cfa-miR-383 | -1.13417532 | 6.33E-20 |
| cfa-miR-3958 | 4.02892198 | 0 |
| cfa-miR-410 | 4.99465289 | 0 |
| cfa-miR-411 | 4.5291066 | 0 |
| cfa-miR-424 | 2.24124918 | 4.20E-33 |
| cfa-miR-429 | 1.23731328 | 0 |
| cfa-miR-432 | 4.68650127 | 0 |
| cfa-miR-433 | 4.61745118 | 1.22E-86 |
| cfa-miR-449a | 4.19807289 | 0 |
| cfa-miR-449b | 6.79269276 | 2.00E-31 |
| cfa-miR-450a | 1.69174739 | 0 |
| cfa-miR-450b | 1.44328647 | 0 |
| cfa-miR-451 | -2.41052798 | 0 |
| cfa-miR-452 | 2.67402649 | 0 |
| cfa-miR-455 | 3.07915907 | 0 |
| cfa-miR-483 | 8.16284446 | 7.50E-09 |
| cfa-miR-485 | 4.9041897 | 4.87E-109 |
| cfa-miR-486 | -2.42803835 | 0 |
| cfa-miR-486-3p | -1.35657557 | 2.02E-05 |
| cfa-miR-487a | 4.88104038 | 1.05E-50 |
| cfa-miR-487b | 4.6691041 | 1.99E-297 |
| cfa-miR-490 | 4.8852332 | 1.77E-241 |
| cfa-miR-493 | 4.01972149 | 8.95E-29 |
| cfa-miR-494 | 5.96183642 | 0 |
| cfa-miR-495 | 4.98573654 | 5.00E-55 |
| cfa-miR-496 | 1.60860904 | 0.04807585 |
| cfa-miR-497 | -1.26376349 | 0 |
| cfa-miR-503 | 2.03495163 | 2.52E-36 |
| cfa-miR-504 | 1.42233195 | 0.00280088 |
| cfa-miR-539 | 4.79268018 | 1.10E-07 |
| cfa-miR-542 | 1.34930883 | 6.04E-251 |
| cfa-miR-543 | 4.5585697 | 5.48E-23 |
| cfa-miR-544 | 3.31871543 | 0.00718848 |
| cfa-miR-551a | -2.15134108 | 2.21E-45 |
| cfa-miR-574 | 1.5546822 | 1.85E-296 |
| cfa-miR-592 | 3.71692418 | 1.16E-19 |
| cfa-miR-660 | 1.03709581 | 3.81E-50 |
| cfa-miR-708 | 2.28348794 | 5.94E-173 |
| cfa-miR-758 | 5.12889216 | 4.78E-173 |
| cfa-miR-802 | 6.8409667 | 0.0005446 |
| cfa-miR-889 | 4.40963325 | 0 |
| cfa-miR-8902 | -7.1770208 | 0.00013001 |
| cfa-miR-9 | 2.59929683 | 5.37E-283 |
| cfa-miR-92a | -1.13766273 | 0 |
| cfa-miR-92b | 2.62581239 | 0 |
| cfa-miR-93 | -1.25063961 | 0 |
| cfa-miR-95 | 1.76824743 | 1.89E-05 |
| cfa-miR-96 | 3.3776943 | 4.86E-83 |
| TRB1 TRB4 | | |
| miR_name | fold-change(log2 TR3/TR7) | p-value |
| cfa-let-7b | 1.33509114 | 0 |
| cfa-let-7c | 1.70847295 | 0 |
| cfa-miR-10a | -2.16847601 | 0 |
| cfa-miR-10b | 2.82950953 | 0 |
| cfa-miR-1185 | 7.27208557 | 8.61E-87 |
| cfa-miR-127 | 5.77888942 | 0 |
| cfa-miR-129 | 1.69417308 | 0.00128087 |
| cfa-miR-133a | 2.04089042 | 0 |
| cfa-miR-133b | 3.08970005 | 1.13E-15 |
| cfa-miR-133c | 2.04089042 | 0 |
| cfa-miR-134 | 4.87986883 | 1.70E-50 |
| cfa-miR-135b | -2.40401166 | 5.37E-22 |
| cfa-miR-136 | 5.64726179 | 0 |
| cfa-miR-138a | -2.87592865 | 9.33E-273 |
| cfa-miR-140 | 6.115041 | 0 |
| cfa-miR-142 | -2.05456881 | 0 |
| cfa-miR-144 | -1.60757426 | 0 |
| cfa-miR-145 | 1.00429295 | 0 |
| cfa-miR-1468 | -1.53019959 | 1.07E-12 |
| cfa-miR-146a | -1.14329804 | 0 |
| cfa-miR-147 | 1.20626702 | 1.30E-25 |
| cfa-miR-148a | 1.35980473 | 0 |
| cfa-miR-149 | 1.77596012 | 1.37E-47 |
| cfa-miR-150 | -2.6191165 | 0 |
| cfa-miR-151 | -1.31222074 | 0 |
| cfa-miR-152 | 1.85815324 | 0 |
| cfa-miR-153 | -1.1893736 | 1.59E-14 |
| cfa-miR-155 | 1.06739225 | 9.17E-200 |
| cfa-miR-15b | -1.66159279 | 0 |
| cfa-miR-16 | -1.75455998 | 0 |
| cfa-miR-181a | -2.05964242 | 0 |
| cfa-miR-181b | -1.61437754 | 0 |
| cfa-miR-182 | 2.26159325 | 0 |
| cfa-miR-183 | 2.12982925 | 2.18E-186 |
| cfa-miR-187 | -2.25143389 | 0.00563711 |
| cfa-miR-190a | 1.40604854 | 1.18E-34 |
| cfa-miR-190b | 2.16726133 | 3.21E-52 |
| cfa-miR-192 | -1.50999763 | 0 |
| cfa-miR-194 | -2.00453252 | 2.70E-209 |
| cfa-miR-195 | -2.37612916 | 0 |
| cfa-miR-199 | 1.6753275 | 0 |
| cfa-miR-203 | 1.26371084 | 1.24E-99 |
| cfa-miR-205 | 2.53815828 | 0 |
| cfa-miR-206 | 7.60644223 | 2.67E-06 |
| cfa-miR-214 | 1.40862456 | 1.57E-267 |
| cfa-miR-218 | -1.39594543 | 2.26E-237 |
| cfa-miR-223 | -3.93306317 | 0 |
| cfa-miR-224 | 2.51187677 | 0 |
| cfa-miR-25 | -1.39477196 | 0 |
| cfa-miR-26a | -1.27633275 | 0 |
| cfa-miR-299 | 5.01266015 | 3.14E-87 |
| cfa-miR-30a | -1.77010703 | 0 |
| cfa-miR-30d | -1.97947814 | 0 |
| cfa-miR-31 | 2.24016719 | 0 |
| cfa-miR-32 | -1.21778109 | 1.27E-43 |
| cfa-miR-323 | 4.4752446 | 1.25E-36 |
| cfa-miR-324 | 1.16368976 | 1.16E-10 |
| cfa-miR-326 | -1.42131647 | 0.00743971 |
| cfa-miR-329b | 6.44967109 | 1.41E-24 |
| cfa-miR-330 | -2.02772458 | 2.01E-18 |
| cfa-miR-335 | 4.69153821 | 0 |
| cfa-miR-33a | 1.7015863 | 5.60E-37 |
| cfa-miR-345 | 1.80758147 | 0.00096317 |
| cfa-miR-34a | -2.41532851 | 0 |
| cfa-miR-34b | 5.35421752 | 0 |
| cfa-miR-34c | 4.83509405 | 0 |
| cfa-miR-362 | 1.03710958 | 7.24E-21 |
| cfa-miR-363 | -1.9567319 | 8.65E-187 |
| cfa-miR-370 | 5.61369436 | 8.37E-89 |
| cfa-miR-371 | -2.96559424 | 3.09E-12 |
| cfa-miR-374a | -1.01392296 | 2.59E-165 |
| cfa-miR-375 | 1.32074568 | 0 |
| cfa-miR-376a | 11.88968648 | 8.86E-109 |
| cfa-miR-376b | 9.40028058 | 4.80E-195 |
| cfa-miR-376c | 8.3263396 | 6.86E-10 |
| cfa-miR-377 | 4.25104005 | 1.48E-09 |
| cfa-miR-378 | 1.93545341 | 0 |
| cfa-miR-379 | 7.58445821 | 5.70E-162 |
| cfa-miR-380 | 5.08035299 | 0 |
| cfa-miR-381 | 5.72522697 | 0 |
| cfa-miR-382 | 6.37324918 | 9.41E-68 |
| cfa-miR-383 | -1.65188382 | 3.74E-48 |
| cfa-miR-384 | 2.48552751 | 0.01297181 |
| cfa-miR-3958 | 4.79360796 | 0 |
| cfa-miR-410 | 6.05540948 | 0 |
| cfa-miR-411 | 5.5453126 | 0 |
| cfa-miR-424 | 1.67910807 | 4.06E-23 |
| cfa-miR-429 | 1.4080824 | 0 |
| cfa-miR-432 | 5.25340746 | 0 |
| cfa-miR-433 | 5.73134746 | 4.79E-97 |
| cfa-miR-449a | 6.07944865 | 0 |
| cfa-miR-449b | 5.80741785 | 3.25E-30 |
| cfa-miR-450a | 1.72308829 | 0 |
| cfa-miR-450b | 1.47969258 | 0 |
| cfa-miR-451 | -1.88154233 | 0 |
| cfa-miR-452 | 2.89235752 | 0 |
| cfa-miR-455 | 3.36119364 | 0 |
| cfa-miR-483 | 8.16284446 | 6.54E-09 |
| cfa-miR-485 | 5.01808599 | 4.17E-111 |
| cfa-miR-486 | -2.39493303 | 0 |
| cfa-miR-486-3p | -2.4498582 | 8.37E-21 |
| cfa-miR-487a | 5.38093456 | 1.62E-53 |
| cfa-miR-487b | 4.59941014 | 1.11E-296 |
| cfa-miR-490 | 5.89959013 | 2.42E-265 |
| cfa-miR-493 | 4.71213613 | 5.69E-32 |
| cfa-miR-494 | 6.62000341 | 0 |
| cfa-miR-495 | 6.22269029 | 1.05E-60 |
| cfa-miR-496 | 3.62369023 | 0.00177964 |
| cfa-miR-497 | -1.41121913 | 0 |
| cfa-miR-503 | 3.15891896 | 1.80E-59 |
| cfa-miR-504 | 3.43682682 | 2.84E-07 |
| cfa-miR-539 | 4.80811022 | 9.64E-08 |
| cfa-miR-542 | 1.58508836 | 6.95644429344475e-321 |
| cfa-miR-543 | 4.57294593 | 3.55E-23 |
| cfa-miR-544 | 6.68887971 | 0.00108985 |
| cfa-miR-551a | -2.10081458 | 8.94E-43 |
| cfa-miR-574 | 1.19546712 | 8.75E-200 |
| cfa-miR-592 | 3.07936609 | 6.07E-17 |
| cfa-miR-708 | 2.23563853 | 1.70E-169 |
| cfa-miR-758 | 5.76477985 | 1.32E-183 |
| cfa-miR-802 | 3.48623246 | 0.00350151 |
| cfa-miR-8859a | 1.26577756 | 7.30E-11 |
| cfa-miR-889 | 5.18707313 | 0 |
| cfa-miR-8902 | -7.87884794 | 4.48E-07 |
| cfa-miR-8908a-3p | -9.58413577 | 1.75E-21 |
| cfa-miR-8908a-5p | -3.74365369 | 0.00063005 |
| cfa-miR-9 | 2.81745884 | 4.60438624072047e-311 |
| cfa-miR-92a | -1.21210742 | 0 |
| cfa-miR-92b | 3.56786589 | 0 |
| cfa-miR-93 | -1.37857814 | 0 |
| cfa-miR-95 | 1.68307942 | 3.41E-05 |
| cfa-miR-96 | 3.35889387 | 3.84E-83 |
